# Control of intestinal bacterial proliferation in regulation of lifespan in *Caenorhabditis elegans*

**DOI:** 10.1186/1471-2180-12-49

**Published:** 2012-03-27

**Authors:** Cynthia Portal-Celhay, Ellen R Bradley, Martin J Blaser

**Affiliations:** 1 Departments of Medicine and Microbiology, New York University School of Medicine, NYU Langone Medical Center, 550 First Avenue, OBV A606, New York, N.Y 10016, USA; 2 Yale University School of Medicine, 333 Cedar Street, New Haven CT 06510, USA

## Abstract

**Background:**

A powerful approach to understanding complex processes such as aging is to use model organisms amenable to genetic manipulation, and to seek relevant phenotypes to measure. *Caenorhabditis elegans *is particularly suited to studies of aging, since numerous single-gene mutations have been identified that affect its lifespan; it possesses an innate immune system employing evolutionarily conserved signaling pathways affecting longevity. As worms age, bacteria accumulate in the intestinal tract. However, quantitative relationships between worm genotype, lifespan, and intestinal lumen bacterial load have not been examined. We hypothesized that gut immunity is less efficient in older animals, leading to enhanced bacterial accumulation, reducing longevity. To address this question, we evaluated the ability of worms to control bacterial accumulation as a functional marker of intestinal immunity.

**Results:**

We show that as adult worms age, several *C. elegans *genotypes show diminished capacity to control intestinal bacterial accumulation. We provide evidence that intestinal bacterial load, regulated by gut immunity, is an important causative factor of lifespan determination; the effects are specified by bacterial strain, worm genotype, and biologic age, all acting in concert.

**Conclusions:**

In total, these studies focus attention on the worm intestine as a locus that influences longevity in the presence of an accumulating bacterial population. Further studies defining the interplay between bacterial species and host immunity in *C. elegans *may provide insights into the general mechanisms of aging and age-related diseases.

## Background

Aging results in alterations in multiple physiologic processes [1]. The identification and measurement of markers of aging to predict lifespan is a major element of aging research [2]. Because the nematode *Caenorhabditis elegans *is genetically tractable, it has become a major model organism for studies of aging [3-5], neurobiology [6,7], cell cycle [8], chemosensation [9], microbial pathogenesis, and host defenses [10-12]. *C. elegans *is particularly suited to studies of aging, since numerous single-gene mutations have been identified that affect *C. elegans *lifespan (AGE genes) [3,4,13,14].

*C. elegans *are free-living nematodes residing in the soil, where they feed on bacteria. In the laboratory, *C. elegans *are normally cultured on a lawn of *Escherichia coli *(strain OP50), on which they feed ad libitum. Although *E. coli *OP50 is considered non-pathogenic for the worms, as *C. elegans *age, the pharynx and the intestine are frequently distended and packed with bacterial cells [15]. This striking phenotype of bacterial proliferation exhibited by old animals, has been hypothesized to contribute to worm aging and demise [15,16]. *C. elegans *grown on bacteria that were unable to proliferate, including those killed by UV treatment or by antibiotics, had much lower rates of intestinal packing and longer lifespan [15], suggesting that bacterial proliferation within the gastrointestinal tract may contribute to the death of the animals. One implication of these findings is that as the worms age, they lose the capacity to control intestinal bacterial proliferation. However, perhaps paradoxically, *C. elegans *has a nutritional requirement for live, metabolically active bacteria, since worms fed on non-viable bacteria appear ill and have diminished fecundity [17].

**Figure 1:**
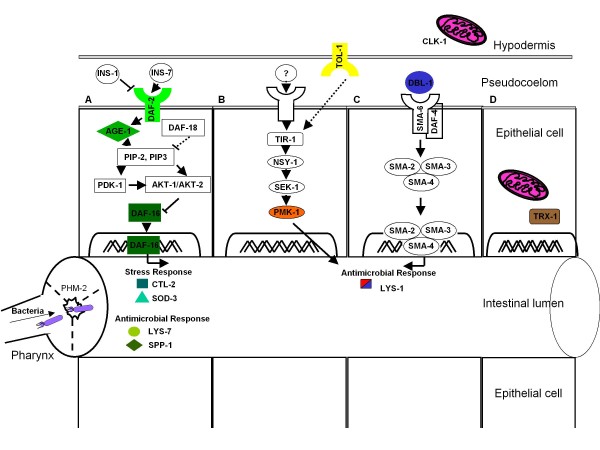
**Signaling pathways important for *C. elegans *intestinal defenses against bacterial proliferation**. A. DAF-2 insulin/IGF-I like signaling pathway. Activation of the DAF-2 receptor results in the phosphorylation of the phosphatidyl inositol 3 kinase (AGE-1) which catalyses the conversion of phosphatidylinositol biphosphate (PiP_2_) into phosphatidylinositol triphosphate (PiP_3_). The kinases PDK-1 and AKT-1/AKT-2 are activated by PiP_3_, which inhibits the transcription factor DAF-16. Relief of this inhibition leads to the expression of a set of stress response and antimicrobial genes. **B. p38 MAPK pathway**. PMK-1 is homologous to the mammalian p38 MAPK and acts downstream of NSY-1/MAKK kinase kinase and SEK-1/MAPK kinase. No interaction between TOL-1 and TIR-1 has been demonstrated. **C. TGF-β pathway**. The TGF-β homologue DBL-1 binds to the heterodimeric receptor SMA-6/DAF-4 and signals through the Smad proteins SMA-2, SMA-3 and SMA-4, which activate the transcription of genes involved in regulation of body size and innate immunity. The expression of lysozyme gene *lys-1 *is under the control of the p38 MAPK pathway and the DBL-1/TGF-β pathway. **D. Mitochondrial enzymes**. CLK-1 is an enzyme required for the biosynthesis of ubiquinoe CoQ9, an acceptor of electrons from both complexes I and II in *C. elegans *cells. Decreased complex I-dependent respiration of *clk-1 *mutants leads to decreased ROS production with lengthening lifespan and slowing development. TRX-1 is a mitochondrial oxidoreductase with important roles in lifespan regulation and oxidative stress response.

*C. elegans *possesses an innate immune system with evolutionarily conserved signaling; anti-microbial innate immunity is modulated by pathways involving the DAF-2 (insulin/IGF-I like) receptor, p38 MAP kinase, and transforming growth factor β (TGF-β) (Figure 1). Aging also substantially diminishes the efficiency of innate immunity [18,19]. We hypothesized that gut immunity is less efficient in older animals, leading to enhanced bacterial accumulation, reducing longevity. To address this question, we evaluated the ability of worms to control bacterial accumulation as a functional marker of intestinal immunity. We considered the effect on longevity of the bacterial species used as nutrient source, as well as host age and host genotype. We studied genes directly related to intestinal immunity and those that are not known to be related. We found a strong inverse relationship between intestinal bacterial accumulation and *C. elegans *longevity, operating across a range of host genotypes. These results suggest that intestinal (commensal) bacterial load is an age and host genotype-related phenotype that can be used to predict *C. elegans *lifespan. By analysis of mutants, we begin to establish a hierarchy of the host immune genes that have greatest effect on the intestinal milieu, and thus on longevity.

## Results

### Role of DAF-2 insulin-signaling pathway on *C. elegans *lifespan

**Figure 2:**
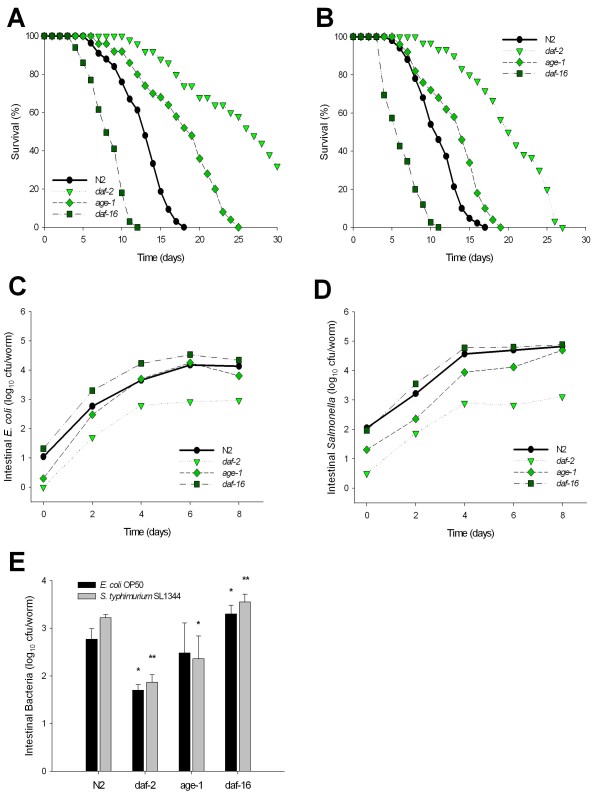
**Density of bacterial accumulation in the *C. elegans *intestine by worm age and genotype, and bacterial strain**. Survival of N2 *C. elegans *and DAF-2 pathway mutants when grown on lawns of *E. coli *OP50 **(Panel A) **or *S. typhimurium *SL1344 **(Panel B)**. Intestinal density of viable *E. coli *OP50 **(Panel C) **or *S. typhimurium *SL1344 **(Panel D) **in N2 *C. elegans *and DAF-2 pathway mutants. **Panel E: **Intestinal load of *E. coli *OP50 (dark bars) or *S. typhimurium *SL1344 (grey bars) within N2 *C. elegans *and DAF-2 pathway mutants on day 2 (L4 stage + 2 days) of their lifespan. Data represent Mean ± SD from experiments involving 30 worms/group. Significant difference (*p *< 0.05) compared to N2 worms exposed to *E. coli *OP50 or *S. typhimurium *SL1344, indicated by * or **, respectively.

Under typical laboratory conditions at 25°C on NGM agar plates with a lawn of *E. coli *strain OP50, a culture of wild type (N2) *C. elegans *has a lifespan of ~ 2 weeks [20]. Lifespans are shorter when lawns are composed of bacteria that are more pathogenic for humans [21]; conversely, host mutations that increase resistance to bacterial infection prolong *C. elegans *lifespan [22]. First, we confirmed [23,24] and extended these observations by analyzing the effect on lifespan of the DAF-2 signaling pathway in *C. elegans *exposed to *E. coli *OP50 or the more pathogenic *S. typhimurium *strain SL1344. We sought to confirm whether under the experimental conditions we used, there is a survival difference for worms grown on lawns of *E. coli *OP50 or *S. typhimurium *SL1344. As expected, the average survival in days (TD_50_) for N2 worms exposed to *S. typhimurium *SL1344 was 10.8 ± 1.37 days, significantly (*p = *0.02) shorter than when exposed to *E. coli *OP50 (12.9 ± 0.51) [23,24] (Table 1). Next, we examined whether we also could find the expected differences in lifespan according to worm genotype. As expected, for both the *E. coli *and *S. typhimurium *strains, lifespan was significantly reduced for the *daf-16 *mutants, but significantly increased for the *daf-2 *and *age-1 *mutants, compared to wild type (Figure 2A and 2B; Table 1). These findings, confirming prior observations [22], indicate the importance to lifespan of both bacterial strain and worm genotype related to intestinal immunity.

**Table 1:** Lifespan and intestinal colonization of *C.elegans *N2 and mutants with growth on *E. coli*or *Salmonella*lawns^a^

		*E. coli *OP50		*S. typhimurium*SL1344	
**Genotype**	**Symbol**	**TD_50_** **(Mean ± SD)**	**Day 2 log_10 _intestinal cfu (Mean ± SD)**	**TD_50_** **(Mean ± SD)**	**Day 2 log_10 _intestinal cfu (Mean ± SD)**

**N2**		**12.93 ± 0.50**	**2.76 ± 0.22**	**10.87 ± 1.37**	**3.22 ± 0.07**
*daf-2*		26.45 ± 1.34^^^^	1.70 ± 0.12^^^^	20.17 ± 0.29^^^^	1.87 ± 0.15^^^^
*age-1*		18.75 ± 0.35^^^^	2.48 ± 0.32	13.70 ± 0.14^^^	2.36 ± 0.48^^^
*daf-16*		8.05 ± 0.38^^^^	3.30 ± 0.19	5.53 ± 0.23^^^^	3.55 ± 0.15^
*lys-7*		9.30 ± 0.74^^^	2.93 ± 0.39	8.83 ± 0.25^^^	3.31 ± 0.28
*spp-1*		9.80 ± 0.59^^^	2.67 ± 0.27	8.70 ± 0.14^^^	3.41 ± 0.23
*sod-3*		11.90 ± 1.01	2.87 ± 0.24	10.93 ± 1.23	3.45 ± 0.25
*ctl-2*		9.48 ± 0.29^^^	2.69 ± 0.18	8.98 ± 0.67^^^	3.88 ± 0.14^^^
*dbl-1*		5.80 ± 0.57^^^^	3.35 ± 0.06	4.75 ± 0.79^^^^	3.86 ± 0.19^^^
*lys-1*		10.00 ± 0.40^^^	2.60 ± 0.22	8.95 ± 0.44^^^	3.12 ± 0.24
*pmk-1*		7.40 ± 0.16^^^^	2.58 ± 0.34	6.10 ± 0.99^^^^	3.71 ± 0.78^^^
*tol-1*		10.53 ± 0.31^^^^	2.81 ± 0.15	8.98 ± 0.79^^^	3.53 ± 0.18^^^
*trx-1*		7.70 ± 0.14^^^^	2.95 ± 0.17	6.83 ± 0.38^^	3.30 ± 0.38

^a ^Worms were age-synchronized by a bleaching procedure. Embryos were placed on mNGM agar plates containing *E. coli *OP50 or *S. typhimurium *SL1344 and incubated at 25°C. The L4 stage was designated as day 0. A total of 100 worms were used per lifespan assay. Bacterial colonization of the intestinal tract was determined at day 2 by washing and grinding 10 worms, and plating worm lysates on MacConkey agar. All assays were performed at least three times

^*p*< 0.05, compared to N2

^^*p*< 0.001, compared to N2

### Bacteria accumulate in the *C. elegans *intestine with aging

As worms age, bacteria accumulate in the intestinal tract [15]. However, quantitative relationships between worm genotype, lifespan, and intestinal lumen bacterial proliferation have not been examined. We hypothesized that intestinal environments that are less favorable for bacterial colonization and accumulation predict longer worm lifespan.

To investigate the relationship of bacterial load to *C. elegans *mortality, we measured the numbers of viable bacteria [colony forming units (cfu)] recovered across the lifespan from the *C. elegans *intestine. As N2 worms grown on an *E. coli *OP50 lawn age, the intestinal load increases from < 10^2 ^*E. coli *cfu/worm on day 0 (L4 stage) to 10^4 ^cfu/worm by day 4 and remains at that level through day 8 (Figure 2C), and at least as far as day 14 when > 50% of worms have died (data not shown). Similar trends were observed when N2 worms were grown on *Salmonella *SL1344 lawns, but colonization reached higher (~10^5 ^cfu/worm) bacterial densities (Figure 2D). Thus, as worms age, bacterial loads rise but reach bacterial strain-specific plateaus, extending until their demise.

**Figure 3:**
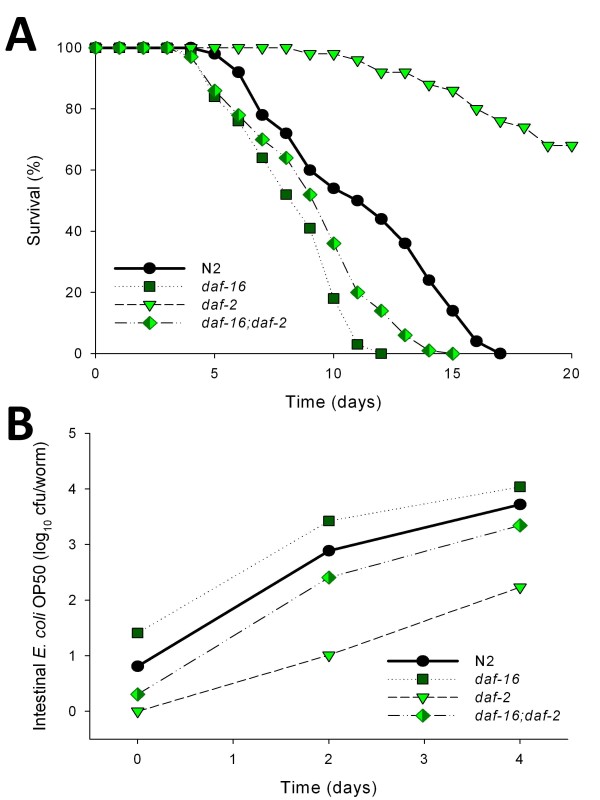
***daf-16 *mutation partially suppresses the *daf-2 *bacterial proliferation phenotypes in *C. elegans***. **Panel A: **Survival of *daf-2*, *daf-16 *single mutants, and *daf-16;daf-2 *double mutant when grown on lawns of *E. coli *OP50. **Panel B: **Intestinal density of viable *E. coli *OP50 in the intestine of the single and *daf-16;daf-2 *double mutants.

We next asked whether bacterial loads are affected by the DAF-2 pathway. The DAF-2 pathway mutants had colonization kinetics paralleling those for N2, but the bacterial loads were often significantly different (Table 1). The long-lived *daf-2 *mutants had about 10-fold lower colonization by both *E. coli *OP50 and *S. typhimurium *SL1344 than did N2 worms (Figure 2E). In contrast, the *daf-16 *mutants had significantly higher densities, consistent with their decreased lifespans. These results suggest a relationship between day 2 colonization levels and ultimate mortality 6-24 days later. Since lifespan extension of *daf-2 *mutants requires the *daf-16 *gene product [14], using the *daf-16(mu86);daf-2(e1370) *double mutant, we asked whether *daf-16 *mutations also would affect the low bacterial loads of *daf-2 *mutants. We confirmed that the *daf-16 *mutation suppresses the lifespan extension of *daf-2 *mutant (Figure 3A), and we now show that it suppresses the low *daf-*2 levels of bacterial colonization as well (Figure 3B).

### Effects of host immunocompromise on bacterial proliferation and lifespan

Next, we asked whether the role of the DAF-2 pathway is unique, or whether other effectors of gut immunity also might play a role in bacterial accumulation. To approach this question, we examined worms with mutations in each of several important pathways in presumed *C. elegans *defenses against intestinal bacteria (see Figure 1). We first studied the p38 MAP kinase pathway by analyzing *pmk-1 *mutants. PMK-1 is the *C. elegans *p38 homologue [25-27], and the p38 MAP kinase cascade is involved in immune defenses to Gram-negative and Gram-positive bacteria, as well as pathogenic fungi [28-30]. Similarly, we studied the DBL-1 pathway using the *dbl-1 *mutant, whose product is homologous to mammalian transforming growth factor-β (TGF-β), and is implicated in pathogen resistance [31,32]. All receptors and Smads from the DBL-1 pathway are strongly expressed in the intestine and/or pharynx of *C. elegans *[33,34]. We also examined mutants in *tol-1*, the only Toll-like receptor (TLR) in *C. elegans*, which is required for the full innate immune phenotype to certain Gram-negative bacteria, for the full expression of ABF-2, a defensin-like molecule expressed in the pharynx [35], and for avoiding pathogenic bacteria [36].

**Figure 4:**
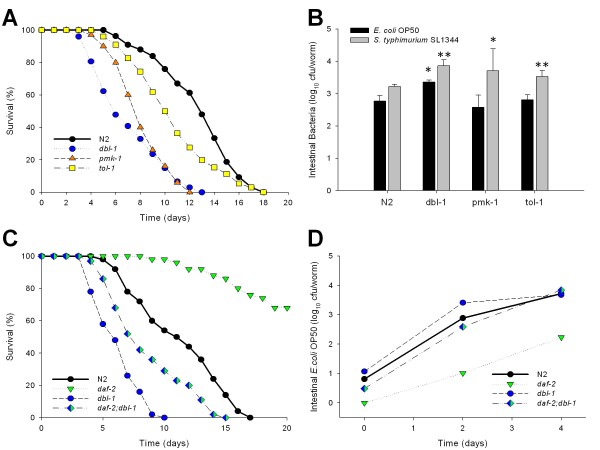
**Survival and density of colonizing bacteria in the intestine of *C. elegans *mutants with altered immune function**. **Panel A: **Survival of N2 *C. elegans *and four mutants with altered intestinal immune function when grown on lawns of *E. coli *OP50. **Panel B: **Intestinal load of *E. coli *OP50 (dark bars) or *S. typhimurium *SL1344 (grey bars) within N2 *C. elegans *and the four mutants with altered intestinal immune function on day 2 (L4 stage + 2 days) of their lifespan. Data represent Mean ± SD from experiments involving 30 worms/group. Significant differences (*p *< 0.05) compared to N2 worms exposed to *E. coli *OP50 or *S. typhimurium *SL1344, indicated by * or **, respectively. **Panel C: **Survival of *daf-2 *and *dbl-1 *single mutants, and the *daf-2;dbl-1 *double mutant when grown on lawns of *E. coli *OP50. **Panel D: **Intestinal density of viable *E. coli *OP50 in the intestine of the single and *daf-2;dbl-1 *double mutants. The *dbl-1 *mutation suppresses both the *daf-2 *intestinal bacterial proliferation and lifespan phenotypes.

The *dbl-1 *mutants showed both markedly reduced lifespan and elevated intestinal bacterial loads (Figure 4A and 4B, and Table 1). In contrast, the *pmk-1 *and *tol-1 *mutants had significantly reduced lifespans, correlating with significantly elevated concentrations of *S. typhimurium *SL1344, although not with intestinal *E. coli *concentrations. These results indicate that across *C. elegans *genotypes, immunocompromise enhances bacterial loads, but is not sufficient to explain lifespan.

Therefore, to examine the interactions between the DBL-1 (TGF-B) and the DAF-2 insulin-signaling pathways, we constructed double mutant worms and analyzed both their longevity and bacterial load. Compared with wild-type N2 strain, *daf-2 *mutants have increased lifespan and lower bacterial load, whereas the opposite was observed for the *dbl-1 *mutants (Figure 4C and 4D). In the *daf-2;dbl-1 *double mutants, there is prolongation of longevity compared with *dbl-1*, with reduction in bacterial load. The phenotypic interaction between the DAF-2 and DBL-1 pathways indicates both playing roles in controlling bacterial load, with consequent effects on longevity.

### Role of downstream immune effector molecules on *C. elegans *longevity and intestinal bacterial load

**Figure 5:**
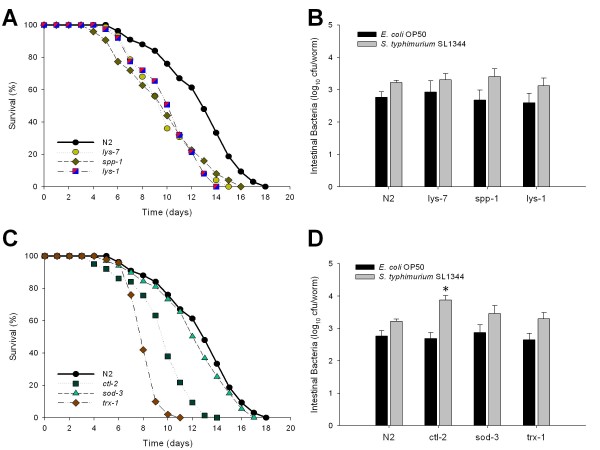
**Role of downstream components of the innate immunity pathways on intestinal bacterial proliferation and *C. elegans *lifespan**. Survival of *C. elegans *mutants with defective expression of antimicrobial peptides **(Panel A) **or oxidative stress enzymes **(Panel C) **when grown on lawns of *E. coli *OP50. **Panel B: **Intestinal load of *E. coli *OP50 (dark bars) or *S. typhimurium *SL1344 (grey bars) with altered intestinal expression of antimicrobial peptides or oxidative stress enzymes **(Panel D) **on day 2 (L4 stage + 2 days) of their lifespan. Data represent Mean ± SD from experiments involving 30 worms/group. Significant (*p *< 0.05) differences in proliferation either *E. coli *or *Salmonella *compared to N2 worms indicated by *.

Since DAF-16 is involved in regulating several antimicrobial proteins and antioxidant enzymes expressed in the intestinal tract [37,38], we next addressed the role of the downstream effector molecules. *C. elegans *has 15 genes that encode lysozymes and 23 genes encoding saposin-like domains, of which *lys-7*, *lys-8 *and *spp-1 *are regulated by the DAF-2 pathway [31,39-41]. Intestinal bacterial loads in *lys-7 *and *spp-1 *mutants were not significantly different from those in N2, but both mutants had significantly decreased lifespan when grown on both the *E. coli *and *Salmonella *lawns (Table 1). For *lys-1*, regulated by both the p38 MAP kinase and TGF-β pathways, mutants have significantly shortened lifespans (Table 1). These results (Figure 5A and 5B; Table 1) indicate the importance of the encoded antimicrobial proteins in regulating lifespan, however, reduction in numbers of colonizing bacteria does not appear to be the sole mechanism for lifespan variation.

When ingesting bacterial cells, *C. elegans *also produce reactive oxygen species (ROS) [42]. The extreme resistance of *daf-2 *mutants to bacterial accumulation may depend on oxidative stress response proteins [42]. To explore this relationship, we studied worms with mutations of *sod-3*, encoding the anti-oxidant superoxide dismutase [43], or of *ctl-2*, a peroxisomal catalase [44]. The *ctl-2 *mutants had significantly decreased lifespan after exposure to either *E. coli *or *Salmonella*, and had significantly higher *Salmonella *density. In contrast, mutations in *sod-3 *had no effect on either lifespan or bacterial load (Figure 5C and 5D; Table 1).

Thioredoxin is involved in maintaining reduced states inside cells [45], and is involved in immune response regulation as well, by controlling NFκB and AP-1 binding [46]. The *C. elegans *thioredoxin (TRX-1) is expressed in neurons and in the intestine [47,48]; recent studies suggest that TRX-1 acts as a fluctuating neuronal signaling modulator within ASJ neurons to monitor the adjustment of neuropeptide expression, including insulin-like proteins, during dauer formation in response to adverse environmental conditions [49]. We found that worms with *trx-1 *mutations have significantly decreased lifespan when grown on *E. coli *or *Salmonella *lawns (Figure 5C; Table 1), and significantly higher bacterial load in late adulthood (see Additional file 1). These studies indicate that control of intestinal bacterial load provides a mechanism to help understand how host tissue oxidative stress responses affect longevity and supports previous observations that neuronal communication mediates longevity control and innate immunity [50-53].

### Distinct colonization patterns according to worm and bacterial genotype are observed in young *C. elegans*

We also considered whether the spatial pattern of intestinal colonization also might affect genotype-specific survival. To address this question, the profile of bacterial accumulation in the gut was examined by considering progressively distal regions of the nematode digestive tract (see Additional file 2A). We found distinct patterns of colonization according to worm and bacterial genotype; for example, colonization of the posterior segments by the *daf-2 *and *ctl-2 *mutant worms was reduced compared with the more anterior segments. However, with worm aging, colonization levels generally equalized and became more homogeneous (see Additional file 2B and 2C). The fluorescence and cfu determinations for day 2 intestinal *E. coli *OP50 and *S. typhimurium *SL1344 concentrations were strongly correlated (see Additional file 2D and 2E). These results indicate that the localization of the large concentrations of cells observed in the intestines may correspond to the large numbers of viable bacteria.

### Relationship between *C. elegans *genotype, colonizing strain, and lifespan

**Figure 6:**
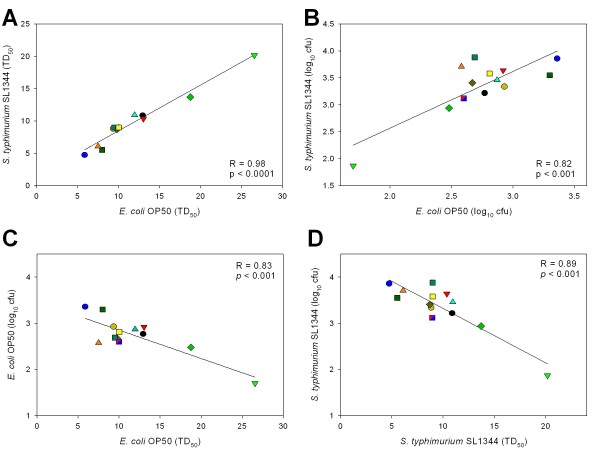
**Relationship between *C. elegans *genotype, colonizing bacterial species, and lifespan**. Symbols for the 14 worm genotypes are as indicated in Table 1. **Panel A: **Relationship of lifespans for worms grown on *E. coli *OP50 and *S. typhimurium *SL1344, measured as TD_50_. Worm survival is strongly correlated with growth on the two organisms (R = 0.98; *p *< 0.0001). **Panel B: **Relationship of intestinal bacterial density for worms grown on *E. coli *OP50 or *S. typhimurium*, measured as day 2 log_10 _cfu. Results show a strong direct correlation for the two bacterial species (R = 0.82; *p *< 0.001). **Panel C: **Relationship between lifespan and intestinal bacterial density for *C. elegans *grown on *E. coli *OP50 lawns. There is an inverse correlation between intestinal bacterial density and survival (R = 0.83; p < 0.001). **Panel D: **Relationship between lifespan and intestinal bacterial density for *C. elegans *grown on *S. typhimurium *SL1344 lawns. There is an inverse correlation between intestinal bacterial density and survival (R = 0.89; p < 0.001).

To assess the biological significance of our observations, we sought to measure how consistent is the pathogenicity of bacterial strains in the lifespan and colonization relationships. The differences in virulence of *Salmonella *and *E. coli *OP50 for *C. elegans*, as reflected in lifespan measurements (Table 1), permitted addressing these questions. Across 12 genotypes related to worm intestinal immunity, lifespan was strongly correlated for the two bacterial strains (R = 0.98; *p *< 0.0001) (Figure 6A). The consistency of these results indicates the importance of host intestinal immunity genotypes in the consequences of the interactions with colonizing bacteria. To address whether intestinal bacterial load was a consistent predictor of lifespan, we assessed survival across worm genotypes, for the two bacterial species examined. First, we found that *E. coli *and *Salmonella *densities were strongly correlated with one another across the studied genotypes related to intestinal immunity (R = 0.82; Figure 6B). For both organisms, there was an inverse correlation between day 2 bacterial density and survival [for *E. coli *OP50 (R = 0.83; Figure 6C), and *S. typhimurium *SL1344 (R = 0.89; Figure 6D)]. These strong relationships suggest that immune handling of bacterial load in the intestine of early adults is an important causative factor in determining lifespan. We chose day 2 to study, because colonization levels were significantly differed amongst the *C. elegans *mutants at that time point (Figure 2E). However we also performed correlations between longevity and bacterial counts for other time points (see Additional file 3), as well as calculations based on a Cox Model, which takes into account bacterial accumulation over time (see Additional file 4). Both results suggest that there exists a significant relationship between longevity and bacterial load throughout early adulthood.

### Relationships between introduced and surviving bacteria in worms with enhanced intestinal immunity

**Figure 7:**
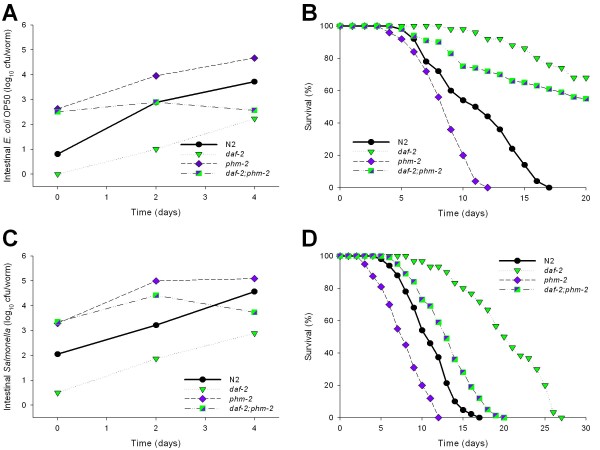
**Density of colonizing bacteria in the intestine of *C. elegans *strains and their survival**. Number (cfu) of *E. coli *OP50 **(Panel A) **or *S. typhimurium *SL1344 **(Panel C) **within the intestine of N2 (wild type), *daf-2 *and *phm-2 *single mutant, and *daf-2;phm-2 *double mutant *C. elegans *strains**. Panel B) **Survival of same strains when grown on lawns of *E. coli *OP50 or *S. typhimurium *SL1344 **(Panel D)**.

The *C. elegans *pharynx contains a grinder that breaks up bacterial cells to provide nutrients for the worm [54]. Grinder-defective worms (e.g. due to *phm-2 *mutation) have shortened lifespan [24]. We hypothesized that the reduced lifespan was related to increased accumulation of viable bacteria in the worm intestine. When grown on an *E. coli *OP50 lawn, the number of viable bacterial cells recovered from the intestine of *phm-2 *mutants was about 10^2 ^*E. coli *cfu/worm at L4 stage (day 0), and increased to 10^4 ^cfu/worm by day 4 (L4 + 4), ~10-fold higher than levels observed in N2 worms (Figure 7A). A similar trend was observed when *phm-2 *mutants were grown on *S. typhimurium *SL1344 lawns, but colonization reached higher bacterial densities, a difference paralleling the other worm genotypes (Figure 7C). After day 4, bacterial concentrations remain on a plateau (data not shown), similar to the observations for the other genotypes.

In lifespan analysis, the TD_50 _for *phm-2 *worms exposed to *E. coli *OP50 (8.7 ± 0.70 days) (Figure 7B), was significantly (*p <*0.001) shorter than for N2 worms (12.9 ± 0.51), and findings were parallel for *Salmonella *(Figure 7D), consistent with prior studies [24]. Thus, the grinder-deficient worms delivered more viable bacteria to the *C. elegans *intestine, and lifespan was reduced compared to N2 for worms grown on either *E. coli *or *Salmonella *lawns.

The long-lived *C. elegans daf-2 *mutants are resistant to bacterial pathogens [22] and as shown above, have significantly lower levels of bacterial colonization (Figure 2, Table 1); these worms have a significantly delayed decline in pharyngeal pumping [2]. Thus, *daf-2 *mutants could be more resistant to bacterial colonization simply because their pharynx remains functional for an extended period of time, or alternatively, because their intestinal milieu is more antimicrobial. To address this question, we constructed *daf-2;phm-2 *double mutants. We found that young *daf-2;phm-2 *double mutants have significantly higher bacterial loads than the wild type and *daf-2 *single mutants, resembling the *phm-2 *single mutants (Figure 7A); thus, early on, the *phm-2 *phenotype dominates. However, as the *daf-2;phm-2 *mutants age, they become increasingly capable of controlling bacterial colonization, with accumulation levels diminishing to the *daf-2 *level. Furthermore, their overall lifespan is very similar to the lifespan of *daf-2 *single mutants when exposed to *E. coli *(Figure 7B). Similar trends, although with a more intermediate phenotype, were observed when the worms were exposed to *Salmonella *lawns (Figures 7C and 7D), indicating that the *daf-2 *phenotypes ultimately become dominant. Thus, in the presence of enhanced intestinal immunity, the number of delivered bacterial cells has no long-term effect on bacterial load or on longevity.

**Figure 8:**
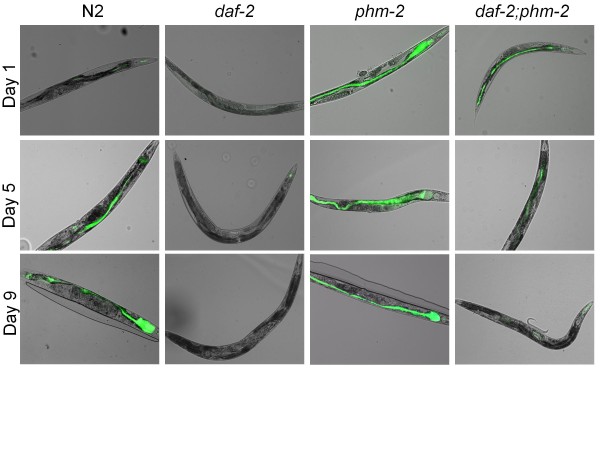
***C. elegans daf-2 *mutants do not require a functional grinder to control intestinal bacterial proliferation**. Fluorescence microscopy of N2, *daf-2 *and *phm-2 *single mutant, and *daf-2;phm-2 *double mutant *C. elegans *strains feeding on GFP-expressing *E. coli*.

To extend these observations, the profile of bacterial accumulation in the intestinal lumen after feeding *E. coli *OP50 expressing GFP was studied. As before, *E. coli *accumulated in the intestine of N2 worms as they aged, leading to a marked distension of the intestinal lumen by day 9 (Figure 8). The *daf-2 *and *phm-2 *single mutants showed contrasting phenotypes, with no bacterial accumulation detected by day 9 and noticeable bacterial packing from day 1, respectively. The kinetics of bacterial accumulation observed in the *daf-2;phm-2 *double mutants correlated with the cfu quantitation (Figure 7C), indicating increasing control of bacterial load over time.

### Relationships between introduced and surviving bacteria in worms with decreased intestinal immunity

**Figure 9:**
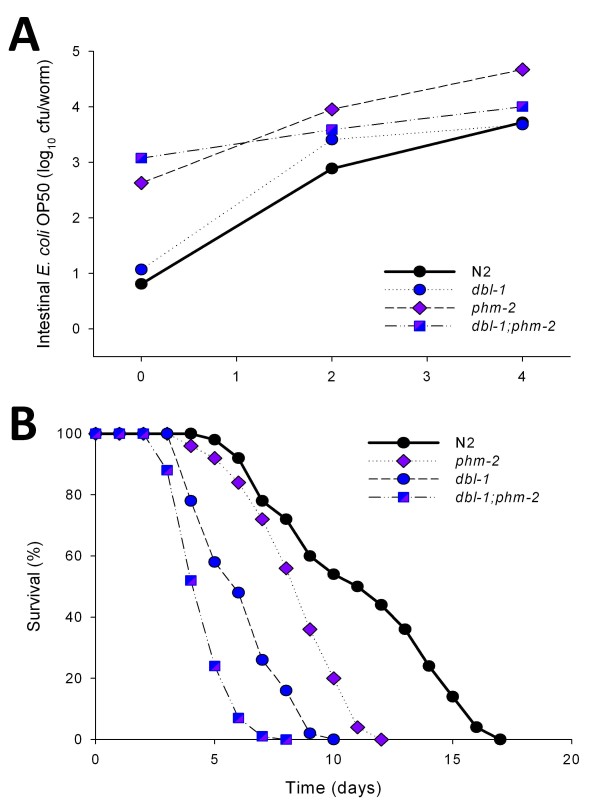
**Immunocompromised *C. elegans *are hypersusceptible to bacterial accumulation**. **Panel A: **Number (cfu) of *E. coli *OP50 within the intestine of N2, *dbl-1 *and *phm-2 *single mutant, and *dbl-1;phm-2 *double mutant *C. elegans *strains**. Panel B: **Survival of same strains when grown on lawns of *E. coli *OP50.

To examine the effect of both increased bacterial delivery to the intestine and decreased immunity, we created a pharynx defective (*phm-2*) and immunocompromised (*dbl-1) *double mutant [31,55]. As before, the *dbl-1 *single mutant showed a difference in bacterial load compared with N2 (Figure 9A), as well as a decreased lifespan reflecting their diminished immunity (Figure 9B). Bacterial load on day 0 (L4 stage) were markedly (100 fold) higher in the *dbl-1;phm-2 *double mutants than in the *dbl-1 *single mutant and N2 wild type worms, and 10 times higher than in the *phm-2 *single mutant (Figure 9A). As worms grew older, they were ill-appearing; by day 3, they had decreased body movement and coordination, decreased pharyngeal pumping, and showed a dramatic reduction in survival (Figure 9B). The bacterial concentrations did not increase as much as the *phm-2 *single mutants, most likely because they were feeding poorly. The early life results indicate that the DBL-1 pathway and the pharynx have additive effects in control of bacterial load, with drastic effects on survival when both are interrupted.

### Effect of mitochondrial function on bacterial proliferation and lifespan

Finally, we asked whether intestinal bacterial load is affected by genes known to have effects on lifespan that are independent of gut immunity. Ubiquinone (coenzyme Q) biosynthesis, essential in mitochondrial respiration, requires demethoxyubiquinone hydroxylase, encoded by *clk-1 *[56]. *C. elegans clk-1 *mutants that generate diminished amounts of reactive oxygen species (ROS) and subsequent reduced levels of oxidative damage [57,58], have prolonged lifespans and resistance to stress induced by UV irradiation, heat, or reactive oxygen [56,59]. Inactivation of *clk-1 *results in an average slowing of a number of developmental and physiological processes, including cell cycle, embryogenesis, post-embryonic growth, rhythmic behaviors, and aging [60]. No role in innate immunity has been described so far.

**Figure 10:**
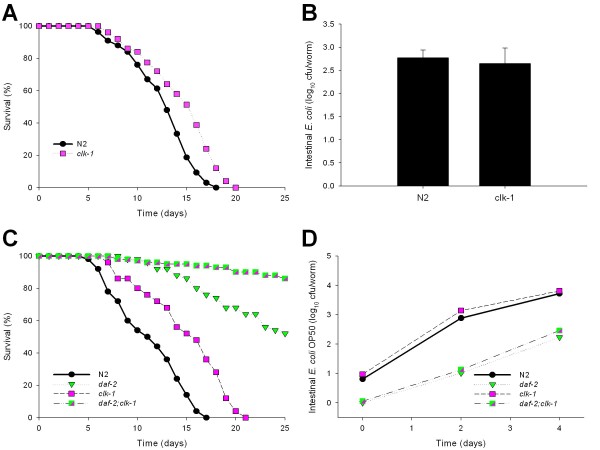
**Daf-2 mutation suppresses the *clk-1 *mitochondrion-dependent intestinal bacterial proliferation phenotype**. Survival of N2 *C. elegans *and *clk-1 *mutants when grown on lawns of *E. coli *OP50 **(Panel A). Panel B: **Intestinal load of *E. coli *OP50 within N2 *C. elegans *and *clk-1 *mutants on day 2 (L4 stage + 2 days) of their lifespan. Data represent Mean ± SD from experiments involving 30 worms/group. **Panel C: **Survival of *daf-2 *and *clk-1 *single mutants and the *daf-2;clk-1 *double mutant when grown on lawns of *E. coli *OP50. **Panel D: **Intestinal density of viable *E. coli *OP50 in the intestine of the *daf-2 *and *clk-1 *single mutants and the *daf-2;clk-1 *double mutants.

As predicted, the *clk-1 *mutants had a prolonged lifespan compared to N2, when grown on lawns of *E. coli *OP50 (Figure 10A).We then assessed whether *clk-1 *affects intestinal bacterial accumulation. We found that the *clk-1 *mutants had intestinal *E. coli *concentrations that were not significantly different from wild type worms (Figure 10B), consistent with the independence of its longevity phenotype on intestinal bacterial accumulation.

Genetic analyses have provided evidence that lifespan extension by *clk-1 *is distinct from the DAF-2 signaling pathway, since *daf-2;clk-1 *double mutants live much longer than either single mutant, and mutations in *clk-1 *cannot be suppressed by *daf-16 *loss-of-function mutations [61]. First, we confirmed that the *daf-2;clk-1 *double mutant has prolonged survival compared to either single mutant (Figure 10C). We next considered the interplay of the *clk-1 *and the *daf-2 *pathways in relation to intestinal bacterial density. We found that the *daf-2;clk-1 *double mutant had intestinal bacterial concentrations that mirror *daf-2 *single mutants (Figure 10D), suggesting *clk-1 *plays no role on intestinal bacterial accumulation. That the double mutant has longer survival than either single mutant (Figure 10C) indicates independence of their longevity mechanisms.

## Discussion

To better understand aging, we studied intestinal bacterial accumulation in *C. elegans *differing in the bacterial species that they ingest, as well as their genotype and maturation. Here, we provide evidence that the extent of intestinal bacterial accumulation early in adulthood, which is controlled by gut immunity that decreases with age, is strongly and inversely correlated with longevity.

Bacteria are the source of nutrition for *C. elegans*, but ultimately as the worms age, viable bacteria accumulate in the intestine [15]. Worms grown on the soil bacterium *Bacillus subtilis *have a longer lifespan compared to those grown on *E. coli *OP50 or many other tested bacterial species [22]. However, worms that are grown on *B. subtilis *spores produce fewer eggs and are smaller and thinner than those fed on vegetative cells of *B. subtilis *or *E. coli *OP50 [62]. This observation indicates that growth on spores compared to vegetative (metabolically active) bacterial cells limits nutrient availability. Thus, vegetative bacteria represent two competing elements to *C. elegans*: a nutrient that fosters development and fecundity, and a toxic component that may reduce lifespan [17]. Worm defenses, including the pharyngeal grinder and intestinal immunity, act to mitigate the latter phenomenon.

The nematode responds to bacteria with conserved innate immune responses, however, aging is accompanied by a decline of immune functions [18,19]. This may represent a general evolutionary process, since after reproductive age individuals compete with their own progeny for available nutrients. Although the functionality of the *C. elegans *immune system during aging has been extensively examined [38,63], we now have simultaneously examined longevity and control of bacterial proliferation across worm genotype, age, and bacterial strain differences. We confirm that viable bacteria accumulate in the *C. elegans *intestine as they age [15], and now show that both bacterial strain type and worm genotype related to gut immunity affect intestinal bacterial accumulation, which might play a significant role in lifespan determination, since we found that lifespan and bacterial load are inversely correlated. Previous studies had quantified bacterial proliferation by CFU enumeration only in N2 worms [64]. More recent studies showed substantially fewer bacteria in the gut of certain long-lived *C. elegans *mutants; however, these observations were by semi-quantitative microscopy only [65]. By quantitatively characterizing the kinetics of bacterial proliferation in the *C. elegans *intestine, in wild type and mutant worms, we establish a basis to better dissect the interplay of bacteria, host genotypes, and age.

One of the aims in this study was to characterize the kinetics of intestinal bacterial colonization. *Salmonella *is a pathogen of *C. elegans *that permits examining this question since it kills worms relatively slowly, rather than in a rapid manner. However, other than consistently higher numbers, there were few cases in which *Salmonella *and *E. coli *results differed greatly. These differ from previous data that reported significant differences in the lifespan of *C. elegans *when grown on *Salmonella *compared to *E. coli *[23]. The discrepancy might be explained in part by differences in methodology, since in this work we grew the worms on lawns of *Salmonella *rather than exposing them as L4's. However, *E.coli *also is pathogenic to *C. elegans *[15,31,64], and many *C. elegans *antimicrobial genes are induced, some even more strongly (*lys-1 *and *spp-1*) than in the presence of other pathogens [40]. As such, *E. coli *is just one other bacterial species to which *C. elegans *can sense and respond.

In our experimental system, we found significant differences in bacterial accumulation at day 2 of adult life, and that variation in the intestinal bacterial loads among the immunodeficient mutants correlated with lifespan differences. Why were differences in bacterial proliferation significant at day 2? One explanation is that since *C. elegans *produces nearly all of its progeny within the first 2 days of its adult life [66], immunity is tightly regulated during development and early adult life, but not post-reproductively. Consistent with this, a striking decrease in expression of PMK-1 regulated genes and a decline in PMK-1 levels in aging animals was recently described [67], suggesting a diminished role for PMK-1 pathway in host defense towards the end of life. Therefore, a decline in immune function in late adult life may either be non-selected, or may be selected at a population level, since as discussed above, non-reproducing worms limit population numbers and stability, since they compete with their progeny for resources [68]. The longevity of *C. elegans *in the wild is substantially (10-fold) shorter than under laboratory conditions [68]; it is probable that most worms die just after laying eggs, since nutrient availability usually is limiting in natural settings.

If the immune system of *C. elegans *experiences an age-related decline [67], which is accompanied by other age-related changes such as pharyngeal deterioration and reduced defecation [69], why does the bacterial load reach a strain-specific (and host-genome-specific) plateau that extends until their demise? One possibility is that a cohort effect exists, in which the fraction of worms examined in late worm adulthood constitutes a subpopulation that survived because they maintain the ability to control bacterial proliferation. Alternatively, late in life the bacterial populations develop specific syntrophic equilibria [70] that are resilient to changes in host milieu.

That the long-lived *daf-2 *mutants resist intestinal bacterial accumulation may be due to enhanced expression of luminal antimicrobial proteins and antioxidant enzymes as evidenced using DNA microarray analysis [38,71-73]. Consistent with this hypothesis, we found that mutants lacking expression of the antimicrobial proteins *lys-7 *and *spp-1*, and the oxidative stress response enzyme *ctl-2 *had diminished lifespan. Since *C. elegans *immune responses generate ROS when bacterial pathogens are ingested [42], oxidative stress responses may aid in resistance by protecting against ROS-induced tissue damage. Thus, antioxidants in the gut protect from oxidative stress, preserving adequate intestinal cell function. The *ctl-2 *mutants also had significantly higher *S. typhimurium *density, consistent with an ROS resistance model. However, the intestinal bacterial densities of *lys-7*, *lys-1*, and *spp-1 *worms were not significantly different from N2. One explanation might be redundancy of the antimicrobial protein genes (15 encoding lysozymes and 23 encoding saposin-like domains) in *C. elegans*. If the numerous genes act in concert, the increased longevity of the *daf-2 *mutants might reflect synergies of individual genes that exert relatively small effects on lifespan and on bacterial colonization. Although the *daf-2 *effect also could reflect reduced senescence of the pharyngeal apparatus or defective pumping, the mixed phenotype of the *daf-2;phm-2 *mutant provides evidence against that hypothesis, and supports the role of enhanced expression of luminal antimicrobial proteins and antioxidant enzymes in controlling bacterial accumulation and ultimately longevity. That the colonization phenotypes of the *daf-2;phm-2 *double mutants is virtually identical to *phm-2 *early in adult life, but with aging, the *daf-2 *effects dominate, indicate the importance of pharyngeal function early in adult life, but that intestinal immune responses dominate as worms become senescent.

Thioredoxin expression may enhance longevity, since transgenic mice expressing human TRX-1 live longer [74]. We confirm that *trx-1 *mutants have significantly decreased lifespan [47,48], and found that intestinal bacterial density was greater in late adulthood (Additional Figure 1) when compared to N2. TRX-1 may affect *C. elegans *longevity and bacterial load due to its antioxidant properties [47], or alternately by modulation of redox-sensitive transcription factors, such as AP-1, that are activated during aging. The fact that bacterial load was greater in late adulthood is consistent with significantly enhanced expression of intestinal TRX-1 expression as worms age [47].

For other effectors of gut immunity, such as those encoded by *dbl-1 *and *pmk-1*, the effects on bacterial load and longevity were strongly inverse. We found that *pmk-1 *mutants have a shorter lifespan than previously reported [75]. Differences in lifespan may be due to different experimental conditions. Troemel et al. added 5-fluorodeoxyuridine (FUDR) to NGM plates seeded with OP50, to prevent *C. elegans *progeny. However, FUDR acts to inhibit DNA synthesis, and also inhibits bacterial proliferation [76]. That abrogating two host anti-bacterial mechanisms (e.g. *dbl-1 *and *phm-2*) produces very short survival indicates synergism between anatomical and immune defenses.

We found a strong correlation between bacterial counts and lifespan. However to better understand the biology of this host-microbial relationship, it would be critical to distinguish between continuing accumulation vs. bacterial proliferation. We address this point in a second manuscript, where we created model systems to evaluate between the possibility of bacterial persistence and proliferation or new bacterial entry [77]. We found that host age as well as bacterial strain determine the nature of bacterial persistence in the *C. elegans *intestine. We also provide evidence for active competition in vivo for colonization sites as well as evidence for in vivo bacterial adaptation. We propose two mechanisms to explain the strong inverse correlation between bacterial load and lifespan. First, the intestinal milieu of older worms is more permissive for bacterial cells in general. Second, over time there is selection for bacteria that are better adapted to the intestinal niche. Our two studies provide support for both mechanisms.

## Conclusions

We performed quantitative studies to determine intestinal bacterial load in *C. elegans *and found a strong correlation between bacterial counts and lifespan. We showed that as adult worms age, they lose their capacity to control bacterial accumulation, and provide evidence that intestinal bacterial load, regulated by gut immunity may play a role in lifespan determination. In total, these studies focus attention on the worm intestine as a locus that influences longevity in the presence of an accumulating bacterial population. Further studies defining the interplay between bacterial species and host immunity in *C. elegans *may provide insights into the general mechanisms of aging and age-related diseases.

## Methods

### *C. elegans *strains and growth conditions

All strains (Table 2) were provided by the *Caenorhabditis *Genetic Center and maintained on modified (0.30% peptone) nematode growth media (mNGM), using standard procedures [78]. The *daf-2;dbl-1 *double mutant was constructed using standard genetic methods [79]. Male stocks were established by heat shock [80] or occurring spontaneously in hermaphrodite populations maintained at 15°. We crossed *daf-2 *males with *dbl-1 *hermaphrodites and F2 animals were picked onto individual plates and grown at 20°C. Presumed double mutants were chosen from plates in which progeny exhibited a *dpy *(fat and short) [81] phenotype, and confirmed by changing the plates to 25°C and screening for dauer larvae [82]. To construct the *daf-2;phm-2 *double mutant, we crossed *daf-2 *males with *phm-2 *hermaphrodites and F2 animals were picked onto individual plates and grown at 25°C. Presumed double mutants were chosen from plates in which progeny were arrested at dauer stage. Double mutants were confirmed by direct microscopic observation of the pharynx (see Additional file 5).

**Table 2:** *C.elegans *single gene mutants used in this study

Strain	Genotype	Function	Relevant *C. elegans *phenotype	Reference*
N2	Wild type		Reference *C. elegans *strain	[20]
*daf-2*	(e1370)III	Insulin-like receptor gene	Extended lifespan, increased resistance to heat, oxidative stress, and pathogens.	[14,22]
*age-1*	(hx546)II	Phosphatidylinositol-3 kinase. Downstream of *daf-2*.	Similar to *daf-2*	[22,83]
*daf-16*	(mu86)I	Fork-head transcription factor. Negatively regulated by the *daf-2 *pathway.	Decreased lifespan, decreased resistance to heat, oxidative stress, and pathogens.	[22,84]
*lys-7*	(ok1384)V	Lysozyme	Induced by *S. marcescens *infection	[31]
*spp-1*	(ok2703)	Saposin-like protein	Active against *E. coli *and expressed in the intestine	[85]
*sod-3*	(gk235)X	Superoxide dismutase	Increased susceptibility to *E. faecalis*	[42]
*ctl-2*	(ok1137)II	Catalase	Decreased lifespan, increased susceptibility to *E. faecalis*	[42,44]
*dbl-1*	(nk3)V	Homologue of mammalian TGF-β	Enhanced susceptibility to pathogens	[31,86]
*lys-1*	(ok2445)	Lysozyme	Induced by *S. marcescens *infection	[31]
*pmk-1*	(km25)	p38 MAP kinase homolog	Enhanced susceptibility to pathogens	[27]
*tol-1*	(nr2033)I	Sole Tol-like receptor.	Unable to avoid pathogenic bacteria. Susceptible to killing by gram negative bacteria. .	[35,36]
*trx-1*	(ok1449)II	Thioredoxin	Decreased lifespan	[47,48]
*phm-2*	(ad597)I	Pharynx morphogenesis	Defective terminal bulb. Allows greater numbers of intact bacteria to enter the intestinal tract.	[54]
*clk-1*	(e2519)III	Coenzyme Q Mitochondrial function	Extended lifespan	[56]

*All strains were provided by the Caenorhabditis Genetic Center, University of Minnesota

### Bacterial strains, plasmids, and growth conditions

*E. coli *OP50 [20] and *S. typhimurium *SL1344 [87] have been described. *S. typhimurium *SL1344 containing plasmid pSMC21 was kindly provided by Fred Ausubel [23]. Cultures were grown in Luria-Bertani (LB) broth at 37°C supplemented or not with ampicillin (100 μg/ml). Bacterial lawns used for *C. elegans *lifespan assays were prepared by spreading 25 μl of an overnight culture of the bacterial strains on 3.5 cm diameter mNGM agar plates. Plates were incubated overnight at 37°C and cooled to room temperature before use.

### Lifespan assays

*C. elegans *lifespan determinations essentially followed established methods [15,23]. However, to avoid competition between introduced bacterial strains, nematodes were age-synchronized by a bleaching procedure [78], then embryos were incubated at 25°C on mNGM agar plates containing *E. coli *OP50 or *S. typhimurium *SL1344. The fourth larval stage (L4) was designated as day 0 for our studies, and worms were transferred daily to fresh plates to eliminate overcrowding by progeny and until they laid no further eggs. Worm mortality was scored over time, with death defined when a worm no longer responded to touch [14]. Worms that died of protruding/bursting vulva, bagging, or crawling off the agar were excluded from the analysis [88]. Kaplan-Meir survival analysis was performed using GraphPadPrism5. For each bacterial lawn, the time required for 50% of the worms to die (TD_50_) for each mutant population was compared to that for the wild type population, using a paired *t *test. A *P*-value < 0.05 was considered significantly different from control. A total of 100 worms were used in each lifespan experiment, and all were performed at least in duplicate.

### Bacterial colonization assay

Nematodes were age-synchronized by bleaching [78], and embryos were incubated at 25°C on mNGM agar plates containing *E. coli *OP50 or *S. typhimurium *SL1344, as above, to prepare for the bacterial colonization assays. Bacterial colonization of *C. elegans *was determined using a method adapted from Garsin et al. [64] and RA Alegado (personal communication and [89]). At each time point tested, 10 worms were picked and placed on an agar plate containing 100 μg/ml gentamicin to remove surface bacteria. They then were washed in 5 μl drops of 25 mM levamisole in M9 buffer (LM buffer) for paralysis and inhibition of pharyngeal pumping and expulsion, then were washed twice more with LM buffer containing 100 μg/ml gentamicin, and twice more with M9 buffer alone. The washed nematodes then were placed in a 1.5 ml Eppendorf tube containing 50 μl of PBS buffer with 1% Triton X-100 and mechanically disrupted using a motor pestle. Worm lysates were diluted in PBS buffer and incubated overnight at 37°C on MacConkey agar. Lactose-fermenting (*E. coli*) and non-fermenting (*Salmonella*) colonies were quantified, and used to calculate the number of bacteria per nematode.

### Fluorescence microscopy

Worms were washed and placed on a pad of 2% agarose in a 5 μl drop of M9 buffer with 30 mM sodium azide as an anesthetic. When the worms stopped moving, a coverslip was placed over the pad and worms were examined by fluorescence microscopy using a Leica DMI 6000B inverted microscope. For comparisons, the nematode digestive tract was divided in three regions of approximately equal length (anterior, middle, posterior) for quantitative studies; bacterial load and location were analyzed using Image-Pro Plus (version 6.0) software.

### Statistical analysis

All assays were performed at least in duplicate. Linear regression analysis was performed using Sigma Plot V.10. Data were analyzed using two-sample *T*-tests assuming equal variances; *p *< 0.05 was considered significantly different from control.

## Authors' contributions

CPC conducted experiments, data/statistical analysis, and manuscript preparation. ERB conducted experiments. MJB provided the conceptual framework, experimental design, and manuscript preparation. All authors read and approved the final manuscript.
